# Correlates of and Barriers to the Utilization of Health Services for Delivery in South Asia and Sub-Saharan Africa

**DOI:** 10.1155/2013/423403

**Published:** 2013-10-28

**Authors:** Nai-Peng Tey, Siow-li Lai

**Affiliations:** Faculty of Economics and Administration, University of Malaya, 50603 Kuala Lumpur, Malaysia

## Abstract

The high maternal and neonatal mortality rates in South Asia and Sub-Saharan Africa can be attributed to the lack of access and utilization of health services for delivery. Data from the Demographic and Health Surveys conducted in Bangladesh, India, Pakistan, Kenya, Nigeria, and Tanzania show that more than half of the births in these countries were delivered outside a health facility. Institutional delivery was closely associated with educational level, family wealth, place of residence, and women's media exposure status, but it was not influenced by women's work status and their roles in decision-making (with the exception of Nigeria). Controlling for other variables, higher parity and younger women were less likely to use a health facility for delivery. Within each country, the poorer, less educated and rural women had higher unmet need for maternal care services. Service related factors (accessibility in terms of cost and distance) and sociocultural factors (e.g., did not perceive the need for the services and objections from husband and family) also posed as barriers to institutional delivery. The paper concludes with some suggestions to increase institutional delivery.

## 1. Introduction

Globally, approximately 287,000 women died from causes related to pregnancy and childbirth in 2010. Of these, 162,000 were in Sub-Saharan Africa and 83,000 were in South Asia. The maternal mortality ratio (MMR defined as the number of women who die during pregnancy and childbirth per 100,000 live births) ranges from 16 in the developed countries to 220 in South Asia and 500 in Sub-Saharan Africa [1]. Lack of access to and utilization of health care services for delivery are among the main reasons for the high maternal and neonatal mortality rates in these regions [2–5]. Maternal death can occur anytime in pregnancy, but delivery is by far the most dangerous time for both mother and baby [6].

The major complications that account for 80 percent of all maternal deaths are severe bleeding and infections after childbirth, high blood pressure during pregnancy and unsafe abortion [7]. Antenatal care, delivery by skilled health professionals, and postnatal care would ensure timely management and treatment of complications to reduce maternal deaths. Despite the importance of institutional delivery in preventing maternal death, about 42 percent of the births in developing countries were delivered outside a health facility, and 35 percent were not attended by trained personnel. Noninstitutional delivery made up more than 80 percent of the births in a few less developed countries such as Ethiopia (95 percent), Afghanistan, Bangladesh, Lao People's Democratic Republic, and Nepal [8].

Factors that prevent women from receiving or seeking health care during pregnancy and childbirth include inadequate services, poverty, distance, lack of information, and cultural practices [7, 8]. Health facilities and services vary widely between the developed and developing countries. In low resource countries, the hospital bed-population and doctor-population ratio was about 0.4 and 0.2 per 1,000 population respectively, while the corresponding figures for the developed countries are 6 and 3 per thousand population. Health expenditure per capita ranges from a mere USD 26.8 in low income countries to USD 224 in middle income countries, USD 382 in upper middle income countries and USD 4,879 in high income countries [9]. 

Numerous studies on the utilization of health facilities have been carried out at the national or subnational level in various parts of the world, including Sub-Saharan Africa and South Asia [10–19]. However, comparative study on the utilization of maternal care services between the two regions is relatively scarce. One particular study that covers the two regions provided the estimates on the number of births in Sub-Saharan Africa and South Asia that will not be attended by a skilled birth attendant between 2011 and 2015 [20]. A cross-country analysis using data from DHS conducted in 31 countries indicates that women's education, economic status, and empowerment are closely associated with the utilization of maternal health services. DHS data from 21 countries in Sub-Saharan Africa show that teenagers in the region have poorer maternal health care than older women with similar background characteristics [21].

The main objective of this paper is to examine the determinants of sociocultural, service and information related barriers to the use of health facilities for childbirth. A better understanding of these barriers is essential for implementing various strategies to increase women's utilization of health facilities to reduce maternal and child deaths.

## 2. Materials and Methods

### 2.1. Data

Data for this study come from the Demographic and Health Surveys (DHS) conducted in 3 selected South Asian countries and 3 African countries in 2006–2010. MEASURE DHS Project has been funded by USAID with contributions from other donors to carry out surveys in developing countries on demographic and health issues that can inform policy. The DHS apply multistage probability sampling to provide nationally representative samples of women of reproductive age (i.e., aged 15–49 years). Since 1984, DHS have been conducted in 85 countries based on a set of core questionnaires to allow comparison across countries. The data are available to researchers through an online database [22].

Bangladesh, India, and Pakistan were chosen to represent South Asia subcontinent, while Kenya, Nigeria, and Tanzania were chosen to represent the Sub-Saharan Africa. These countries were selected based on the population size and the availability of DHS data for the most recent period—2007 for Bangladesh, 2006 for India, 2007 for Pakistan, 2009 for Kenya, 2008 for Nigeria, and 2010 for Tanzania.

The proportion of women who had more than one birth in the five years prior to the survey ranged from about one-quarter in Bangladesh to one-third in India and 45–52 percent in the other four countries in this study. This analysis is based on the most recent birth within the reference period.

### 2.2. Statistical Methods

Place of delivery, type of birth attendants, and reasons for not using a health facility for the delivery are the dependent variables for this study. For logistic regression analysis, place of delivery and type of birth attendant were recoded into binary variables, taking the value 1 for institutional delivery, 0 otherwise, and 1 for delivery by trained personnel, 0 otherwise. 

The independent variables may be classified as individual-level variables (educational level of women and husband, maternal age, media exposure, women's work status, and their status in the family); household-level variables (family income or wealth); and community-level variables (urban-rural residence). In previous studies, education, household socioeconomic status, and urban-rural residence are consistently significant predictors of service utilization, while all other variables are less consistent predictors across studies [2, 10–12, 16, 23–38].

Household income data were not collected in DHS. Instead, the data sets contain a variable on the household's quintile classification of wealth index generated through principal component analysis based on household ownership of various assets and on housing characteristics. Description of the construction of this variable can be read from the report for each country.

Women's status is represented by a variable on whether a woman has a final say on her own health care (Yes = 1, No = 0).

Media exposure is an index based on the following:

- frequency of reading newspaper or magazine (more frequent = 1/less frequent = 0);
- frequency of listening to radio;
- frequency of watching television.

Women who scored 0 to 1 were grouped as a “Low” exposure to the media and 2 to 3 were grouped as a “High” exposure to the media.

We began with a description of the sample distribution for each independent variable, followed by the distribution of place of delivery and the type of birth attendant for each country. The independent variables were interrelated with one another, with confounding effects on delivery care. For instance, family wealth index was closely associated with the educational level of women and their husband; higher educated women tended to marry higher educated men; and educational level and financial status were also closely associated with media exposure and birth parity. Binary logistic regression analyses were used to examine the odds of using health facilities and services for delivery within the multivariate context. Each of these variables represents a different construct, and the problem with multicollinearity is not a concern.

Odds ratio of value greater than 1 shows that the likelihood of the occurrence of an event is higher in a particular group as compared to the reference group, and vice versa. Odds ratio of less than 1 is deducted from 1 and interpreted as a percent less likely. For instance, an odds ratio of 0.8 is interpreted as 20 percent less likely for the occurrence of an event as compared to the reference group.

## 3. Results

### 3.1. Characteristics of the Samples

The sample for this study was based on the last birth of currently married women aged 15–49 years who had given birth 5 years preceding the survey.  Table 1 summarizes the total sample for each country and the percentage distribution by the independent variables.

The level of urbanization in all the six countries was considerably lower than the average for the less developed world which stood at 46 percent in 2010. A significant proportion of women and their husbands in these countries had never been to school. Gender gap in education was most pronounced in Pakistan, where two-thirds of the women had never been to school. Women in the three Sub-Saharan African countries, especially in Tanzania, had very high labor force participation rate. In contrast, few women in South Asia were reported as working. 

In the five countries where data are available, women in Kenya had relatively high status in the family as compared to the rest. In contrast, Tanzanian women had the lowest status within the family. Women in all the six countries in this study had low media exposure.

For the wealth quintile, the deviation from 20 percent for the various subgroups can be explained by the uneven distribution of childbearing women in the 5 years preceding the survey. In Kenya, Nigeria, and Pakistan, women giving births in the five years before the survey were over-represented by those in the poorest wealth quintile, but they were over-represented by those in the richest quintile in Bangladesh and India.

The modal age group was below 30 years for all the six countries, with Bangladesh and India having the youngest age structure. Except for Bangladesh and India, the number of women having 1-2, 3-4, and 5 or more children was rather evenly split.

### 3.2. Place of Delivery and Birth Attendance

**Figure 1:**
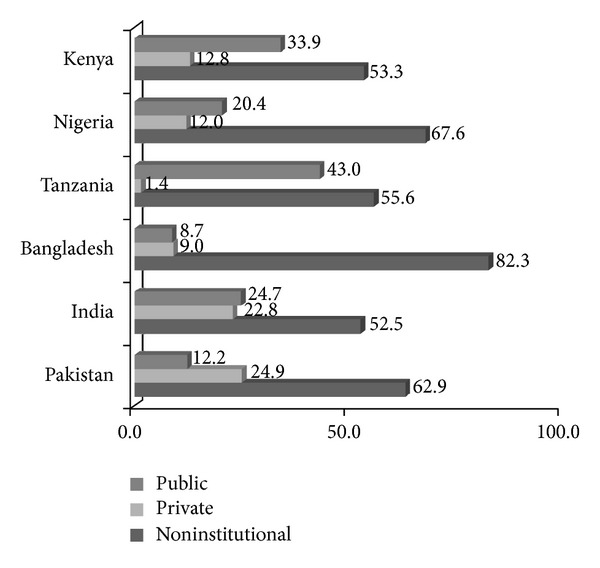
Percentage distribution of the place of delivery by country.

In all the six countries, more than half of the births occurred outside a health facility, and most of these were home delivery.  Figure 1 shows that noninstitutional delivery was highest in Bangladesh, followed by Nigeria and Pakistan. Of those who delivered in a health facility, women from the three Sub-Saharan African countries were much more likely to deliver in a public health facility rather than a private health facility. In Bangladesh and India, about the same proportion of women used the public and private health facilities but Pakistani women were twice as likely to use a private health facility rather than a public health facility for childbirth. 

**Figure 2:**
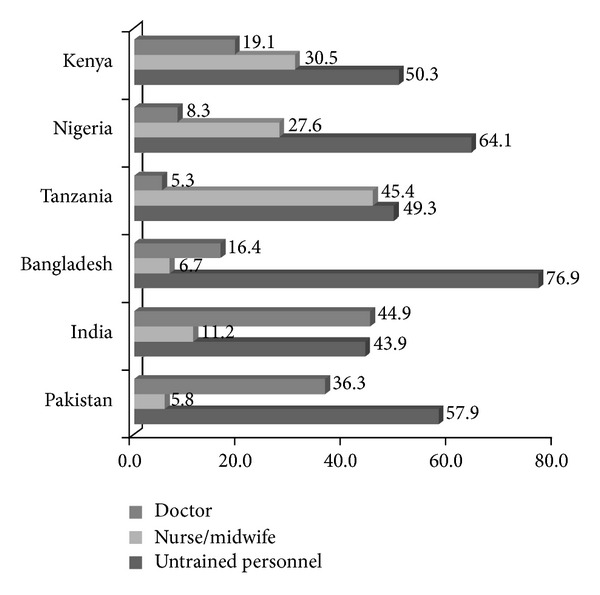
Percentage distribution of the type of birth attendant by country.

Use of trained attendant for delivery corresponded closely with the place of delivery. All the births that occurred in a hospital or clinic were delivered by a trained attendant. A sizable proportion of home delivery was attended by trained personnel, and this ranged from 5-6 percent in Bangladesh, Kenya, and Nigeria to 15–17 percent in Tanzania and India. The proportion of births attended by an untrained attendant, including traditional midwives, was highest in Bangladesh (76.9 percent) and lowest in India (46.9 percent). There was no discernible difference in births attended by untrained personnel between South Asia and Sub-Saharan Africa. In South Asia, most of the deliveries by trained attendants were conducted by doctors, but nurses were the main birth attendants in the three Sub-Saharan African countries (Figure 2).

### 3.3. Determinants of the Use of Health Facilities and Services for Delivery

Logistic regressions were used to examine the determinants of the use of health facilities for childbirth in the multivariate context (Table 2). In all the six countries under study, place of residence, educational level of women and their husbands, wealth index, women's exposure to media, maternal age, and birth parity had significant effects on the use of a health facility for delivery. In Tanzania and India, rural women were only half as likely as urban women to deliver in a health facility, but the urban-rural effect was much smaller in Nigeria, where rural women were 24 percent less likely than urban women to give birth in a health facility.

Utilization of health facilities for delivery also varied widely by region within each country, probably due to the uneven distribution of hospitals, health centers, and clinics, with concentration in the more developed regions. More detailed tabulations of DHS data show that women from the more developed regions were much more likely than those from the less developed region to deliver in a health facility. In all countries, rural women were much less likely than urban women to use a health facility for delivery (11.6 percent versus 26.5 percent in Bangladesh, 18.2 percent versus 99.3 percent in India, 21.2 percent versus 44.6 percent in Pakistan, 21.2 percent versus 88.6 percent in Kenya, 8.3 percent versus 70.7 percent in Nigeria, and 22.9 percent versus 72.1 percent in Tanzania).

In all the six countries, women who had never been to school were least likely to have institutional delivery, while those with at least secondary education were most likely to do so. The educational effect on institutional delivery was weakest in Tanzania. In Nigeria and all the three countries in South Asia, the effect of the wife's education on the use of a health facility for childbirth was much stronger than that of the husband's education.

The odds of using a health facility for delivery were about the same for both working and nonworking women in Tanzania and the three South Asian countries. In these four countries, higher educated women were less likely to work as compared to their lesser educated counterparts. In Kenya and Nigeria, where working women had higher odds of institutional delivery, higher educated women were more likely than lesser educated women to be currently working.

With the exception of Nigeria, there was no significant difference in institutional delivery between women who had a say in their own health care and those who did not have a say. In Nigeria, women with no say were significantly less likely to use a health facility for delivery. Contrary to expectation, Indian women who did not have a say in their health care were a little more likely than those who have a say to deliver in a health facility.

Except for Bangladesh, the odds of institutional delivery decreased monotonically from the richest quintile families to the poorest quintile families. In India and Nigeria, women from the poorest quintile families were 86–90 percent less likely than those from the richest quintile families to give birth in a health facility. In all the five countries where data are available, women with low media exposure were much less likely than those with high media exposure to give birth in a health facility.

Of the two demographic control variables, birth parity was a more important predictor of the use of a health facility for childbirth as compared to maternal age. Lower parity women were much more likely than higher parity and older women to deliver in a hospital or clinic. This suggests that the more experience a woman had in childbirth, the less likely she would use a health facility for delivery. However, controlling for other variables in the model, younger women were less likely than older women to use the health facilities for delivery.

The determinants in the utilization of trained personnel for delivery corresponded rather closely to that of the place of delivery, as shown in Table 3. In India, where the odds of using a health facility for delivery was not significantly different between working and nonworking women, nonworking women were less likely than working women to have a trained birth attendant.

Family wealth index provides consistently the sharpest differentials in the odds for delivery by a trained attendant across all the six countries. Poorest women in Nigeria were 92 percent less likely to have their births attended by a trained attendant, and in India where the differential was smallest, the corresponding figure was 74 percent. Place of residence, educational level of women and their husbands, and birth parity are significant variables in predicting delivery by a trained attendant, and the findings are in congruence with that of the place of delivery. 

### 3.4. Reasons for Not Using Health Facilities and Skilled Birth Attendants for Delivery

Except for Bangladesh, DHS in the other five countries collected information on the reasons for not delivering in a hospital or clinic. The mean number of responses ranged from 1.03 in Tanzania to 1.3 in Pakistan.  Table 4 shows that the reasons for not using the health facility for delivery varied widely across countries. A high proportion of women in Nigeria and Pakistan (more than half) and India (more than two-thirds) thought that it was not necessary to deliver in a hospital or clinic. This finding corroborates with a study in Indonesia where the preference for traditional birth attendants was strongly affected by traditional belief [39].

Several studies found that women living far away from a health facility were much less likely to have a skilled attendant and an institutional delivery [4, 17]. In this study, distance and lack of transport were the most important reason for the nonuse of health services for delivery in Kenya and Tanzania and the second most important reason in Nigeria.

High cost was the second most often stated reason for the nonuse of health services in India and Pakistan. However, it was of lesser concern to women from the three African countries, especially Tanzania. 

It is notable that a rather sizable proportion of nonusers of health services in Kenya mentioned abrupt delivery, and more than one in ten in Nigeria reported that it was not customary to give birth in a health facility. Other barriers to institutional delivery include objection from husband/family (especially in Pakistan), no facility, and lack of trust in the facility. Only a small proportion mentioned nonavailability of female health provider for not delivering at a health facility. 

More detailed tabulations of data show the very wide variations of reasons for the nonuse of health facility for delivery by region and ethnicity within each country. For instance, in India the proportion of respondents who mentioned “cost too much” ranged from none in Kerala to 48 percent in Bihar; the percentage not using a health facility for delivery because of distance ranged from 4.7 percent in Delhi to 75 percent in Kerala; family objections ranged from none in Kerala to 17.5 percent in West Bengal and “not necessary to use” ranged from 25 percent in Kerala to more than three-quarters in a number of districts.

The ethnic differentials in the reasons for not using a health facility for delivery were most striking in Pakistan, as shown in Table 5. The percentage that did not use the health services for delivery because of high cost, objection from husband/family, and “not necessary” ranged from none to 100 percent. It is noteworthy that all Potowari women did not use a health service for delivery due to the objection from husband or family.

## 4. Discussion

Between 1990 and 2010, maternal mortality ratio (MMR) declined by 64 percent in Southern Asia and 41 percent in Sub-Saharan Africa. The MMR in these two regions remains the highest in the world, and it appears unlikely for Sub-Saharan Africa to achieve the target under MDG 5 to reduce the MMR by three-quarters between 1990 and 2015. Because the majority of maternal deaths occur just before, during, or just after delivery, often from complications that cannot be predicted, institutional delivery can reduce the risk of complications and death of mother and baby significantly. Nonetheless, a very high proportion of births in Sub-Saharan African and Southern Asia are occurring outside a health facility and are not delivered by a skilled attendant. Concerted efforts must be made to increase the utilization of maternal care services to achieve the MDG goals in the two regions.

Consistent with the findings of previous research [2, 10, 12, 23, 24, 27, 37, 38, 40], our analysis shows that in all the six countries in this study, women's education, household wealth, and urban-rural residence had the most significant and consistent effects on the utilization of health services for delivery. Higher education is generally associated with urban living, higher income, and better exposure to the media, all of which affect the use of health facilities for childbirth [2, 10, 12, 27, 31, 32, 35, 41]. Our findings corroborate with the findings of previous studies on these aspects.

Urban-rural differentials in health care utilization were due to the concentration of health infrastructure and personnel in urban areas [42]. There is a need for alternative strategies to reach those living in remote areas, including the use of mobile units. 

Although primary school enrolments have increased dramatically in Sub-Saharan Africa and South Asia, these regions are still lagging behind in education. Of the 72 million out-of-school children worldwide, nearly half reside in Sub-Saharan Africa [43]. Less developed countries need to invest more in education and give equal opportunities to the girls and the lower socioeconomic groups. Investing in education will facilitate gender equity and women's empowerment and their labor force participation. Educational improvement will bring about a rise in income level, which in turn will lead to increased utilization of health services towards achieving the MDG goal of improving maternal health. The experience of low-resource Ethiopia in putting three million more children in school than in 2000 with a rural school construction programme and abolition of primary school fees could serve as a good lesson for others [43].

The family wealth index was found to be the most important predictor of the use of institutional delivery. Hence, the high cost of health services (of much concern in India and Pakistan) and the inability of the poor to pay would pose as a serious barrier to the use of health facilities for delivery. Programs and strategies aimed at removing financial barriers in some countries have been found to be effective in increasing the utilization of delivery care services [44, 45].

Past studies found that women who had a say in their own health care were more likely to use a health facility for health care, including delivery [33, 34, 46, 47]. In Yemen, underutilization of modern delivery care was attributed to women's low autonomy and status [25]. Contrary to these studies, our findings show that whether or not a woman had a say in her own health care had little effect on institutional delivery. 

Lack of exposure to media also posed as a barrier to the utilization of maternal and child health services [16]. Our finding suggests that the nonuse of a health facility could probably be due to the lack of knowledge or information on the importance of giving birth in a health facility and the location of such facilities. The low media exposure among women in Sub-Saharan Africa and South Asia could be partly due to their low educational level and the lack of media facilities and reports. Hence, concerted efforts should be made to use the mass media more effectively to disseminate the benefits and importance of institutional delivery and the risks of not using these services. Reproductive health education should be incorporated into the school curriculum. Countries may also learn from the successes of the community-based safe motherhood intervention in Tanzania that has proven to be very effective in promoting the utilization of obstetric care and a skilled attendant at delivery [17]. Users of health services could be encouraged to serve as agents to motivate others in their own community to make use of health facilities for delivery.

The likelihood of institutional delivery decreased with the number of children, as women may feel more confident and feel that there is no need for institutional delivery. There is therefore a need to inform women of the increased risk of the complications of higher order pregnancies and older maternal age and to encourage them to continue using the health services for subsequent births. 

Barriers to the use of health facilities for delivery varied widely across and within a country. Service related factors such as cost (not affordable), distance/lack of transport, and availability were the main barriers to institutional delivery in Kenya and Pakistan, while sociocultural factors, especially the perception that there was no need to use the health services for delivery, were the main reasons for noninstitutional delivery in India, Nigeria, and Tanzania. Hence, appropriate strategies need to be implemented to remove these barriers by the respective countries to reduce the unmet need for services for specific target groups, especially the poor and those living in remote areas. 

Cultural beliefs and practices and the lack of awareness and knowledge often pose as barriers to the utilization of health services for delivery [4, 15, 17, 23, 36, 48–51]. Many women and their husbands may not realize the various risk factors associated with pregnancy and delivery. More information, education, and motivation programs and campaigns should be held to reach out to the public, including the males. 

The private sector plays a very important role in maternal care services, especially in Pakistan and India. However, services provided by the private sector were not so accessible to the poor because of the higher cost. Hence, there is a need to form a strong public-private partnership in delivering the health services. Private hospitals and doctors should be encouraged to play their role in fulfilling their corporate social responsibilities. Besides, the government could consider providing some forms of incentives such as tax rebate and subsidies to private doctors to make their services more readily available and accessible to the poor. More efforts should be made to engage the private hospitals and doctors in the national health programs.

More than half of the births in South Asia and Sub-Saharan Africa are delivered by traditional birth attendants and other untrained attendants. Delivery by untrained attendant is probably the main reason for the high maternal mortality in the regions, as traditional birth attendants are neither ready to handle complications during and after delivery nor do they have the necessary equipment and medicine to treat hemorrhage (uncontrolled bleeding), sepsis (infection), and hypertensive disorders, which are the main causes of maternal death. The services of traditional birth attendants will continue to be sought after in the foreseeable future, and hence, there is a need to give them the necessary support and equip them with the necessary skills, including basic knowledge and information on HIV/AIDS. Further, traditional birth attendants should be encouraged and given some incentives to refer their clients to the hospitals and clinics. 

A previous study found that young women who initiated antenatal care were more likely to use skilled professional assistance at delivery than their counterparts who initiated antenatal care later [28]. Women should be informed of the importance of initiating antenatal checks during the first trimester and informed of the importance and benefits of institutional delivery during their antenatal visits. 

Of the six countries in this study, Bangladesh had the lowest hospital bed-population ratio at 3 per 10,000 population, while Kenya and India had a relatively higher ratio at 14 and 9 per 10,000 population, respectively [9]. There is therefore a need for the governments to allocate more resources to the health sector to make health services widely accessible, including the remote rural areas, and to train and recruit more health personnel. The governments can consider giving free maternal care, as in the case of Ghana [44], provide vouchers such as in Cambodia [45], or make other arrangements to promote institutional delivery among the poor. In some countries, increasing ambulance services may be necessary to bring patients to the health facilities, as many had cited lack of transportation as the main reason for not using a health facility for delivery.

## 5. Conclusion 

Despite making substantial progress towards improving maternal health, many countries in South Asia and Sub-Saharan Africa are still grappling with the problems of high maternal mortality and are struggling to achieve the Millennium Development Goals (MDG) to reduce the MMR by three-quarters by 2015. Given the low institutional delivery and high maternal mortality in the regions, there is a need to target the groups who do not use health services for delivery and address the barriers that exist. Besides removing the service-related barriers, public health information and education need to be widely disseminated to influence the public opinion on the benefits and the importance of health care utilization. The successes and good practices of some countries in implementing various policies and programs to increase institutional delivery could serve as models for other developing countries. The effective implementation of programs and strategies directed at specific target groups requires the involvement of various stakeholders to remove the barriers to the utilization of maternal care services.

## Figures and Tables

**Table 1:** Percentage distribution of the study population by country.

Variables	Kenya	Nigeria	Tanzania	Bangladesh	India	Pakistan
Sample size	3,365	17,025	4,510	4,811	36,115	5,655
Place of residence						
Rural	74.2	73.3	80.0	64.6	60.5	65.0
Urban	25.8	26.7	20.0	35.4	39.5	35.0
Wife's education						
None	19.9	50.9	24.7	25.4	38.2	66.5
Primary	55.4	22.1	62.7	30.5	14.2	13.8
Secondary+	24.7	27.1	12.6	44.1	47.6	19.6
Husband's education						
None	15.2	41.4	17.5	31.7	22.6	36.5
Primary	47.5	20.7	67.4	28.3	14.4	16.2
Secondary+	37.3	37.8	15.0	40.0	63.1	47.3
Women's work						
Not working	44.9	35.2	17.9	76.0	71.0	76.1
Working	55.1	64.8	82.1	24.0	29.0	23.9
Final say on own health care						
No	30.3	59.3	43.6	39.1	35.9	—
Yes	69.7	40.7	56.4	60.9	64.1	—
Wealth index						
Poorest	26.5	26.7	18.5	19.1	16.6	22.4
Poorer	16.9	23.6	21.6	19.9	17.5	21.6
Middle	16.4	19.1	20.5	18.5	20.1	19.6
Richer	16.8	16.6	21.7	19.2	22.1	18.6
Richest	23.4	14.0	17.6	23.4	23.7	17.9
Media exposure						
Low	70.4	75.9	81.1	83.3	69.0	—
High	29.6	24.1	18.9	16.7	31.0	—
Age						
<30	57.7	52.2	51.5	73.5	70.2	52.9
30–39	34.4	35.7	36.2	23.3	26.9	37.7
40+	7.9	12.2	12.4	3.2	2.9	9.4
Birth parity						
1-2	37.8	31.6	33.1	57.9	57.7	35.3
3-4	31.5	29.0	30.1	28.3	27.4	28.4
5+	30.7	39.5	36.8	13.9	14.9	36.2

**Table 2:** Logistic regression on “using health facility for delivery in the past 5 years” by selected variables.

Variables	Kenya	Nigeria	Tanzania	Bangladesh	India	Pakistan
	Odds ratio	Odds ratio	Odds ratio	Odds ratio	Odds ratio	Odds ratio
Place of residence						
Rural	0.59 (0.4, 0.8)	0.77 (0.7, 0.9)	0.50 (0.4, 0.6)	0.56 (0.5, 0.7)	0.51 (0.5, 0.5)	0.69 (0.6, 0.8)
Urban (reference)	1.00	1.00	1.00	1.00	1.00	1.00
Wife's education						
None	0.37 (0.3, 0.5)	0.24 (0.2, 0.3)	0.69 (0.5, 0.9)	0.21 (0.1, 0.3)	0.43 (0.4, 0.5)	0.43 (0.4, 0.5)
Primary	0.56 (0.4, 0.7)	0.54 (0.5, 0.6)	0.91 (0.7, 1.2)	0.45 (0.4, 0.6)	0.65 (0.6, 0.7)	0.60 (0.5, 0.7)
Secondary+ (reference)	1.00	1.00	1.00	1.00	1.00	1.00
Husband's education						
None	0.36 (0.3, 0.5)	0.60 (0.5, 0.7)	0.61 (0.5, 0.8)	0.57 (0.4, 0.8)	0.89 (0.8, 1.0)	0.78 (0.7, 0.9)
Primary	0.68 (0.6, 0.8)	1.06 (1.0, 1.2)	1.03 (0.8, 1.3)	0.56 (0.4, 0.7)	1.05 (1.0, 1.1)	0.83 (0.7, 1.0)
Secondary+ (reference)	1.00	1.00	1.00	1.00	1.00	1.00
Women's work						
Not working	0.83 (0.7, 1.0)	0.70 (0.6, 0.8)	0.94 (0.8, 1.1)	1.12 (0.9, 1.4)	1.03 (1.0, 1.1)	1.02 (0.9, 1.2)
Working (reference)	1.00	1.00	1.00	1.00	1.00	1.00
Final say on own health care						
No	1.00 (0.8, 1.2)	0.64 (0.6, 0.7)	0.93 (0.8, 1.1)	0.96 (0.8, 1.2)	1.07 (1.0, 1.1)	—
Yes (reference)	1.00	1.00	1.00	1.00	1.00	—
Wealth index						
Poorest	0.18 (0.1, 0.3)	0.10 (0.1, 0.1)	0.28 (0.2, 0.4)	0.33 (0.2, 0.5)	0.14 (0.1, 0.2)	0.18 (0.1, 0.2)
Poorer	0.29 (0.2, 0.4)	0.17 (0.2, 0.2)	0.32 (0.2, 0.4)	0.22 (0.2, 0.3)	0.20 (0.2, 0.2)	0.25 (0.2, 0.3)
Middle	0.44 (0.3, 0.6)	0.30 (0.3, 0.4)	0.44 (0.3, 0.6)	0.33 (0.2, 0.4)	0.33 (0.3, 0.4)	0.32 (0.3, 0.4)
Richer	0.51 (0.4, 0.7)	0.51 (0.4, 0.6)	0.53 (0.4, 0.7)	0.46 (0.4, 0.6)	0.49 (0.5, 0.5)	0.51 (0.4, 0.6)
Richest (reference)	1.00	1.00	1.00	1.00	1.00	1.00
Media exposure						
Low	0.68 (0.6, 0.8)	0.83 (0.8, 0.9)	0.80 (0.7, 1.0)	0.67 (0.6, 0.8)	0.67 (0.6, 0.7)	—
High (reference)	1.00	1.00	1.00	1.00	1.00	—
Age						
<30	0.61 (0.4, 0.9)	0.58 (0.5, 0.7)	0.60 (0.5, 0.8)	0.45 (0.2, 0.9)	0.73 (0.6, 0.9)	0.65 (0.5, 0.9)
30–39	0.87 (0.6, 1.2)	0.88 (0.8, 1.0)	0.82 (0.7, 1.0)	0.84 (0.4, 1.7)	1.06 (0.9, 1.3)	0.81 (0.6, 1.0)
40+ (reference)	1.00	1.00	1.00	1.00	1.00	1.00
Birth parity						
1-2	2.56 (1.9, 3.4)	1.83 (1.6, 2.1)	2.33 (1.9, 2.9)	4.40 (2.7, 7.3)	3.71 (3.4, 4.1)	1.99 (1.7, 2.4)
3-4	1.75 (1.4, 2.2)	1.35 (1.2, 1.5)	1.51 (1.3, 1.8)	2.18 (1.3, 3.6)	1.67 (1.5, 1.8)	1.26 (1.1, 1.5)
5+ (reference)	1.00	1.00	1.00	1.00	1.00	1.00

Constant	8.76	8.08	4.43	0.84	3.33	4.08

Figures in brackets show 95 percent confidence intervals.

**Table 3:** Logistic regression on “using trained personnel in the past 5 years” by selected variables.

Variables	Kenya	Nigeria	Tanzania	Bangladesh	India	Pakistan
	Odds ratio	Odds ratio	Odds ratio	Odds ratio	Odds ratio	Odds ratio
Place of residence						
Rural	0.64 (0.5, 0.8)	0.68 (0.6, 0.8)	0.37 (0.3, 0.5)	0.49 (0.4, 0.6)	0.54 (0.5, 0.6)	0.66 (0.6, 0.8)
Urban (reference)	1.00	1.00	1.00	1.00	1.00	1.00
Wife's education						
None	0.46 (0.3, 0.6)	0.23 (0.2, 0.3)	0.59 (0.4, 0.8)	0.28 (0.2, 0.4)	0.44 (0.4, 0.5)	0.46 (0.4, 0.6)
Primary	0.53 (0.4, 0.7)	0.50 (0.5, 0.6)	0.85 (0.7, 1.1)	0.43 (0.3, 0.5)	0.63 (0.6, 0.7)	0.64 (0.5, 0.8)
Secondary+ (reference)	1.00	1.00	1.00	1.00	1.00	1.00
Husband's education						
None	0.54 (0.4, 0.8)	0.59 (0.5, 0.7)	0.59 (0.5, 0.8)	0.55 (0.4, 0.7)	0.83 (0.8, 0.9)	0.77 (0.7, 0.9)
Primary	0.66 (0.5, 0.8)	1.08 (1.0, 1.2)	1.03 (0.8, 1.3)	0.55 (0.5, 0.7)	0.97 (0.9, 1.0)	0.80 (0.7, 1.0)
Secondary+ (reference)	1.00	1.00	1.00	1.00	1.00	1.00
Women's work						
Not working	0.89 (0.8, 1.1)	0.67 (0.6, 0.7)	0.96 (0.8, 1.2)	1.21 (1.0, 1.5)	0.94 (0.9, 1.0)	0.95 (0.8, 1.1)
Working (reference)	1.00	1.00	1.00	1.00	1.00	1.00
Final say on own health care						
No	1.12 (0.9, 1.3)	0.66 (0.6, 0.7)	0.79 (0.7, 0.9)	0.91 (0.8, 1.1)	1.05 (1.0, 1.1)	—
Yes (reference)	1.00	1.00	1.00	1.00	1.00	—
Wealth index						
Poorest	0.21 (0.1, 0.3)	0.08 (0.1, 0.1)	0.24 (0.2, 0.3)	0.26 (0.2, 0.4)	0.14 (0.1, 0.2)	0.18 (0.1, 0.2)
Poorer	0.29 (0.2, 0.4)	0.15 (0.1, 0.2)	0.27 (0.2, 0.4)	0.19 (0.1, 0.3)	0.18 (0.2, 0.2)	0.25 (0.2, 0.3)
Middle	0.43 (0.3, 0.6)	0.26 (0.2, 0.3)	0.38 (0.3, 0.5)	0.31 (0.2, 0.4)	0.29 (0.3, 0.3)	0.33 (0.3, 0.4)
Richer	0.51 (0.4, 0.7)	0.46 (0.4, 0.5)	0.50 (0.4, 0.7)	0.44 (0.4, 0.6)	0.45 (0.4, 0.5)	0.54 (0.4, 0.7)
Richest (reference)	1.00	1.00	1.00	1.00	1.00	1.00
Media exposure						
Low	0.66 (0.5, 0.8)	0.83 (0.7, 0.9)	0.78 (0.6, 1.0)	0.62 (0.5, 0.8)	0.65 (0.6, 0.7)	—
High (reference)	1.00	1.00	1.00	1.00	1.00	—
Age						
<30	0.60 (0.4, 0.9)	0.62 (0.5, 0.7)	0.50 (0.4, 0.7)	0.53 (0.3, 1.0)	0.81 (0.7, 1.0)	0.64 (0.5, 0.8)
30–39	0.92 (0.7, 1.3)	0.94 (0.8, 1.1)	0.75 (0.6, 0.9)	0.94 (0.5, 1.8)	1.12 (1.0, 1.3)	0.82 (0.7, 1.0)
40+ (reference)	1.00	1.00	1.00	1.00	1.00	1.00
Birth parity						
1-2	2.49 (1.9, 3.2)	1.85 (1.6, 2.1)	3.11 (2.5, 3.9)	3.33 (2.2, 5.1)	3.45 (3.1, 3.8)	2.07 (1.7, 2.5)
3-4	1.69 (1.4, 2.1)	1.36 (1.2, 1.5)	1.84 (1.5, 2.2)	1.62 (1.1, 2.4)	1.67 (1.5, 1.8)	1.25 (1.1, 1.5)
5+ (reference)	1.00	1.00	1.00	1.00	1.00	1.00

Constant	8.96	12.79	10.20	1.76	6.31	5.39

Figures in brackets show 95 percent confidence intervals.

**Table 4:** Percent citing each reason for not delivering at a health facility by country.

Reason for not delivering at a health facility	Kenya	Nigeria	Tanzania	India	Pakistan
Cost too much	13.8	9.6	6.6	22.1	38.6
Facility not open	5.4	5.2	1.0	3.5	5.8
Too far/no transport	45.1	23.5	33.7	12.2	6.6
Do not trust facility	3.0	2.0	1.5	2.6	3.9
No female provider	1.7	1.3	1.0	1.1	0.8
Husband/family did not allow	1.6	4.6	1.9	5.0	7.2
Not necessary	22.3	54.9	19.2	69.6	54.1
Not customary	2.0	11.5	8.1	5.3	6.6
Abrupt delivery	16.1	—	—	—	5.5
Other	5.1	6.9	30.4	3.6	1.9

Mean number of reasons	1.16	1.20	1.03	1.25	1.31

**Table 5:** Percent of women citing each reason for not delivering at a health facility by ethnicity, Pakistan.

Reason for not delivering at a health facility	Cost too much	Too far	Husband/family did not allow	Thought it was not necessary
Ethnicity				
Urdu	31.3	3.0	5.1	57.6
Punjabi	31.6	3.3	3.0	68.4
Sindhi	48.0	5.7	5.9	50.8
Pushto	31.8	6.5	13.3	42.1
Balochi	44.0	19.9	11.6	48.1
Barauhi	32.1	8.6	16.0	59.3
Siraiki	46.5	7.5	5.0	57.0
Hindko	43.2	3.4	3.4	47.7
Kashmiri	0.0	0.0	0.0	100.0
Pahari	25.0	25.0	0.0	25.0
Potowari	100.0	0.0	100.0	0.0
Marwari	56.3	3.1	0.0	50.0
Farsi	0.0	0.0	0.0	66.7
Other	53.2	10.5	5.6	46.0
